# Label-Free Direct Detection of miRNAs with Poly-Silicon Nanowire Biosensors

**DOI:** 10.1371/journal.pone.0145160

**Published:** 2015-12-28

**Authors:** Jing He, Jianjun Zhu, Changguo Gong, Jiming Qi, Han Xiao, Bin Jiang, Yulan Zhao

**Affiliations:** 1 School of Life Science, East China Normal University, Shanghai, PR China; 2 Shanghai Integrated Circuit Research & Development Center, Shanghai, PR China; 3 Ocean Research Center of Zhoushan, Zhejiang University, Zhejiang Province, PR China; 4 Wuhan Medical and Health Center for Women and Children, Wuhan, Hubei Province, PR China; International Centre for Genetic Engineering and Biotechnology, ITALY

## Abstract

**Background:**

The diagnostic and prognostic value of microRNAs (miRNAs) in a variety of diseases is promising. The novel silicon nanowire (SiNW) biosensors have advantages in molecular detection because of their high sensitivity and fast response. In this study, poly-crystalline silicon nanowire field-effect transistor (poly-SiNW FET) device was developed to achieve specific and ultrasensitive detection of miRNAs without labeling and amplification.

**Methods:**

The poly-SiNW FET was fabricated by a top–down Complementary Metal Oxide Semiconductor (CMOS) wafer fabrication based technique. Single strand DNA (ssDNA) probe was bind to the surface of the poly-SiNW device which was silanated and aldehyde-modified. By comparing the difference of resistance value before and after ssDNA and miRNA hybridization, poly-SiNW device can be used to detect standard and real miRNA samples.

**Results:**

Poly-SiNW device with different structures (different line width and different pitch) was applied to detect standard Let-7b sample with a detection limitation of 1 fM. One-base mismatched sequence could be distinguished meanwhile. Furthermore, these poly-SiNW arrays can detect snRNA U6 in total RNA samples extracted from HepG2 cells with a detection limitation of 0.2 μg/mL. In general, structures with pitch showed better results than those without pitch in detection of both Let-7b and snRNA U6. Moreover, structures with smaller pitch showed better detection efficacy.

**Conclusion:**

Our findings suggest that poly-SiNW arrays could detect standard and real miRNA sample without labeling or amplification. Poly-SiNW biosensor device is promising for miRNA detection.

## Introduction

MicroRNAs (miRNAs) are a class of highly conserved non-coding RNA with 19–25 nucleotides. MiRNAs can regulate gene expression through directly binding to 3’ UTR of mRNA or coding sequence[1, 2]. They are involved in various physiological and pathological processes such as cell differentiation, proliferation and apoptosis[3, 4]. Dysregulation of miRNAs are related with cancers[5], atherosclerosis[6] and other diseases. The level of miRNAs in serum or other body fluid have been found to alter in a variety of disease, indicating its potential value for diagnosis and prognosis[7, 8]. However, the clinical application of miRNAs is limited due to their characteristics such as low level, small size and sequence similarity among various members.

Current methods to detect miRNAs are mainly divided into two groups: methods based on amplification or hybridization. The representative of the former is quantitative real-time PCR (qPCR). The later includes northern blotting, *in situ* hybridization, microarray and deep sequencing[9]. Actually, these methods also need sample amplification before hybridization. In general, all the methods mentioned above do not match perfectly with the requirements of clinical detection, such as easy utility, fast response, low cost, high sensitivity and specificity. To date qPCR is considered as the golden standard for miRNAs quantification[10, 11]. But the amplification procedures are time-consuming and qPCR results rely on fluorescent tags labelled during reaction. Deviation may occur during signal acquisition in this indirect method. Low sensitivity and complicated labelling procedures respectively make northern blot and *in situ* hybridization difficult to be routine detection methods for miRNAs[12, 13]. Microarray and deep sequencing are limited to high expense, long reaction time and complicated data analysis[14, 15]. Other methods such as surface plasmon resonance and Raman spectroscopy are challenging because of expensive instruments. Electrochemical biosensors are restricted to its detecting speed and crowding effect of probe.

Silicon nanowire (SiNW) biosensors have advantages such as label-free detection, high sensitivity, rapid response and good selectivity[16]. Zhang *et al* first reported SiNW biosensors could detect 1 fM miRNA directly with peptide nucleic acid (PNA) probe[17]. Dorvel *et al* were able to achieve 100 fM detection level of miR-10b with using SiNW and hafnium oxide dielectrics, and claimed a theoretical limit of 1 fM[18]. Importantly, they took single-stranded DNA (ssDNA) as probe for cost-saving. Notably, miRNAs detected in these studies are standard samples. Here we introduced our poly-SiNW biosensors to detect both standard and real miRNA sample with ssDNA as probes. Since polysilicon is the major materials used in commercial manufacture of SiNW, our study could provide experimental evidences for poly-SiNW application for miRNA detection. Furthermore, we selected nine representative silicon nanowire structures which were different from nanowire width and pitch, the width between two parallel nanowires. The detection efficacy of different structures was compared finally.

## Methods and Materials

### Materials

All chemical reagents were purchased from Sigma-Aldrich and solutions were prepared with Milli-Q water. The sequences of ssDNA and miRNAs were listed in S1 Table and they were synthesized by Invitrogen. Total RNA was extracted from culture HepG2, a liver cancer cell line.

### Fabrication of SiNW biosensors

The structure of poly-SiNW biosensors includes the following layers from down to top: a Si (100) substrate, a SiO_2_ insulating layer, a polysilicon layer and a structural layer formed on the polysilicon layer. Notably, the polysilicon layer comprised a patterned silicon nanowire array. The thickness of the SiO_2_ insulating layer and polysilicon layer was in a range of 1000 Å-5000 Å and 50 Å-1000 Å, respectively. The line width and thickness of the silicon nanowire was in a range of 5 nm-130 nm and 5 nm-100 nm, respectively.

To achieve the above structure, the manufacturing method included the following steps: (1)to provide a Si (100) substrate; (2) to form a SiO_2_ insulating layer on the Si substrate by wet oxidation; (3) to develop a polysilicon layer on the SiO_2_ insulating layer by Low Pressure Chemical Vapor Deposition (LPCVD); (4) to dope polysilicon layer by ion implant; (5) to pattern the polysilicon layer to form a silicon nanowire array by dry etching; (6) to form a SiO_2_ passivation layer on the silicon nanowire by CVD technology.

### Modification of poly-SiNW biosensors

Before modification, the silicon chip was cleaned with ethanol and isopropyl alcohol to remove contaminants. Silanation was carried out by reaction with 2% APTES in 95% ethanol solution for 2 h to convert surface silanol groups to amines. Then the device was washed for three times with absolute ethanol alcohol. After treatment of 1.25% glutaraldehyde for 1 h, ssDNA bind to the aldehyde-modified SiNW surface. After that, the device was washed for three times with Milli-Q water. The poly-SiNW surface was then incubated with 100 nM let-7b probe (Invitrogen) in 1×SSC at room temperature (RT) overnight. Then the surface was washed with 1×SSC for three times to remove unreacted probe[17].

### Sample preparation and MiRNA-ssDNA hybridization

A series of standard samples of let-7b, let-7c and mismatch miRNA sequences were prepared by adding miRNA powders (Invitrogen) in 0.01×SSC solution. Total RNA samples were extracted from cultured human liver cancer cell line HepG2 using Trizol (Invitrogen) by standard protocol. After ssDNA probe binding to SiNW surface, samples were incubated with poly-SiNW chips and miRNA-ssDNA hybridization took place in 0.01×SSC for 1 h at RT. Then the chips were washed with 0.01×SSC for three times.

### Resistance detection and data analysis

Voltage-current curves of each sample were measured before (**Resistance, R**_**0**_) and after hybridization (**Resistance, R**) in Cascade probe station (Cascade Microtech). Changes in resistances reflect hybridization efficiency. Data are analyzed through comparing changes in resistances. After preliminary screening, we found the current-voltage curves (I-V curves) were commonly not straight lines, suggesting resistant of each sample was not a constant. Hence, we analyzed the resistance changed by the following process: (1) to obtain the current-voltage function (cubic function is suitable according to our experience) according the raw curve, which is defined as “*f*(I) = aU^3^+bU^2^+cU+d”; (2) to obtain the conductance-voltage function by derivation “*f*(G) = dI/dU = 3aU^3^+2bU^2^+c”; (3) to calculate the area under curve (AUC) of the conductance-voltage curve in a certain range (0–5 v in this study) “$\int_{0}^{5}f(G)$” to indicate the conductance of each sample; (4) to calculate the resistance change (R/R_0_) by the following equation “$\frac{R}{R_{0}}\equiv \frac{\int_{0}^{5}f(G_{0})}{\int_{0}^{5}f(G)}$”.

Here, U is the voltage; I is the current; G_0_ is the conductance of a biosensor before sample loading; G is the conductance of this biosensor after miRNA sample hybridization; and a, b, c and d are constants.

Every experiment was repeated for at least five times. Measurements were expressed as mean±SD from at least 5 samples. The differences in different groups were compared by one-way ANOVA using SPSS 19.0 for Windows (SPSS, Chicago, IL). Significant difference was defined as probability value P <0.05.

## Results

### Device fabrication and chemical modification

**Fig 1:**
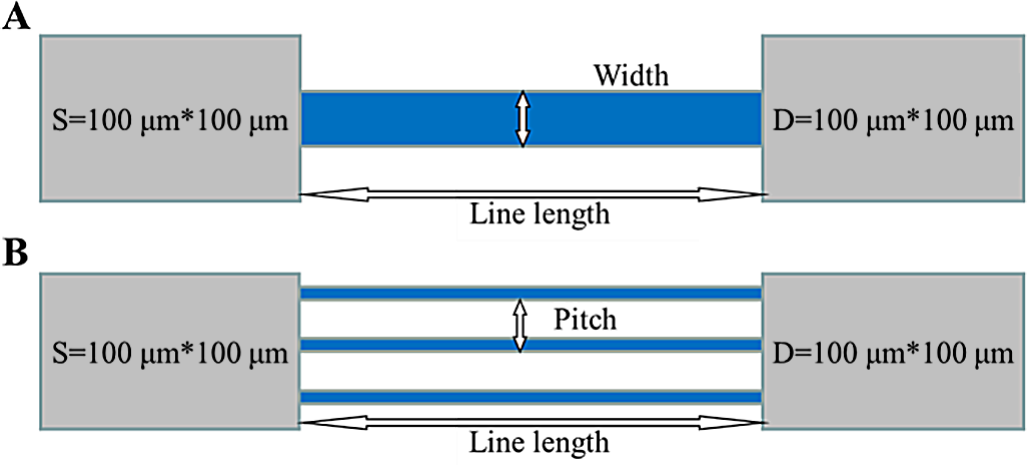
Structures of SiNW biosensor. S: Source, D: Drain. SiNW length is 100 μm. Pitch is width sum of silicon nanowires and lateral blank area. A: Schematic diagram of RR17, RR19 and RR20; B: Schematic diagram of FF47, FF49, FF50, FF57, FF59 and FF60.

The poly-SiNW biosensors used in the work were produced using a top–down Complementary Metal Oxide Semiconductor (CMOS) wafer fabrication based technique, including conventional optical lithography, etching and oxidation. As shown in S1 Flie, a typical 12-inch wafer was divided into 70–80 chips, while one single chip was comprise of about 400 individual biosensors. The fabrication process formed a thin oxide on the poly-SiNW surface and the bulk SiO_2_ outside the poly-SiNW surface. The enhanced sensing behavior was caused by the high surface-to-volume ratio. Each biosensor was designed as different line width (25–300 nm), line length (50–200 μm) and pitch (1–3 μm or no pitch) (Fig 1). After preliminary screening of the 400 different structures of biosensor, 9 structures were selected to run the following experiments because of their stable electronic performance, including FF47, FF49, FF50, FF57, FF59, FF60, RR17, RR19 and RR20 (S2 Table). A representative photo of SiNW through transmission electron microscopy was shown in S1 Fig, demonstrating good topograph feature of poly-SiNW. S2 File showed the surface of poly-SiNW was successfully modified by a series of chemistry reactions. For the final electronic detection, a good biosensor should present a smooth voltage-current curve by electronic test, whereas a failed biosensor did not present a perfect curve because of the broken nanowires (S3 File).

### Detection of miRNA standard sample

**Fig 2:**
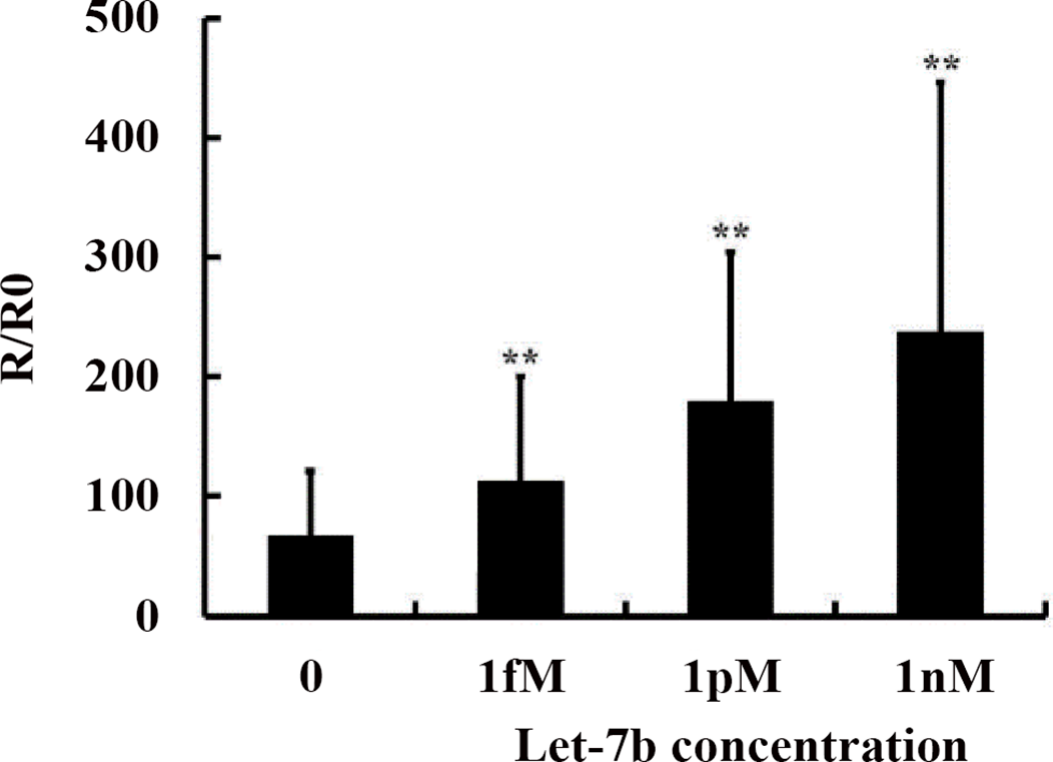
Detection of microRNA standard sample. Let-7b standard sample: 1 nM, 1 pM, 1 fM and 0. Probe: 100 nM Let-7b probe. The detection limit for Let-7b standard sample is 1 fM.

Different concentrations of miRNA were tested with poly-SiNW biosensors. The resistance change before and after DNA-miRNA hybridization primarily depends on the amount of charge layer contributed by miRNA. The more the target miRNA molecules hybridized, the more negative charges accumulated on the SiNW surface, thus the higher the resistance increase. Fig 2 showed the resistance change fold was increased in a concentration-dependent pattern. Evidently, an obvious resistance increase was obtained even when 1 fM let-7b was hybridized to the DNA-functionalized SiNW device. This indicates that ultralow concentrations of miRNA can effectively be detectable with the poly-SiNW device without labeling and amplification.

**Fig 3:**
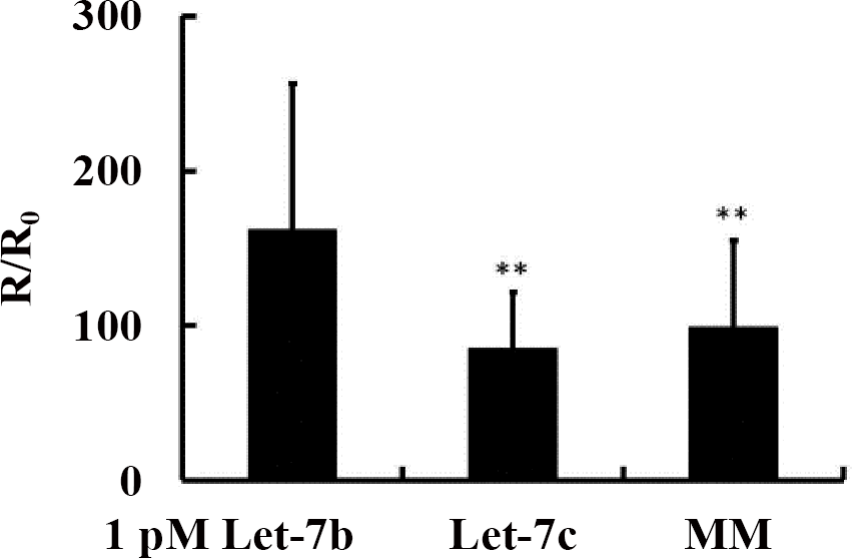
Detection of one-base mismatched microRNA standard sample: 1 pM Let-7b, 1 pM Let-7c and 1 pM mismatch (MM). Probe: 100 nM Let-7b probe. Single base difference between Let-7b and Let-7c is significantly identified at 1 pM concentration level.

The detection specificity of the poly-SiNW device was examined using standard samples of let-7b, let-7c and noncomplementary miRNA sequences. As compared to let-7b, the resistance change caused by let-7c and mismatch miRNA sequences were much lower (Fig 3). The significant difference between let-7b and let-7c demonstrated this poly-SiNW device could even distinguish single base mismatch at the level of 1pM. The high specificity suggests that the poly-SiNW biosensors allow for label-free discrimination between the fully matched and mismatched miRNAs, offering unique advantage over other technologies which require labeling and additional tags.

### Detection of snRNA U6 in total RNA extracted from HepG2 cells

**Fig 4:**
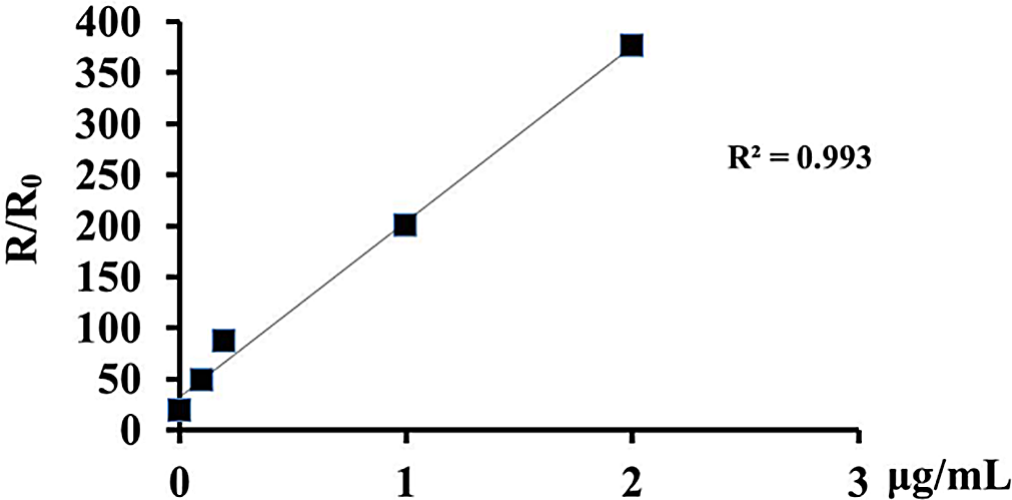
Detection of snRNA U6 in total RNA from HepG2 in five concentrations. There is a well linear relationship among a series of concentrations.

Besides standard miRNA samples, we further detected snRNA U6 in total RNA extracted from HepG2 cells. U6 is commonly considered as internal control in studies of miRNAs. Five concentrations (2, 1, 0.2, 0.1, 0 μg/mL) of total RNA are selected for detection. The results showed a linear relationship between concentration and resistance change with a R^2^ value of 0.993 (Fig 4). Thus, the resistance changes were increased along with the concentration. In addition, this poly-SiNW device can at least detect snRNA U6 in total RNA of 0.2 μg/mL (Table 1).

**Table 1:** Comparison of different pitches in three total RNA concentration (2, 0.2 and 0 μg/mL).

	total RNA	2 μg/mL	0.2 μg/mL	0	T Test, p value		
		mean (SD)	mean (SD)	mean (SD)	2 μg/mL vs 0.2 μg/mL	2 μg/mL vs 0	0.2 μg/mL vs 0
Pitch	FF47-RR20	376 (858)	87 (219)	19 (46)	0.0337	0.0082	0.0121
1 μm	FF47-50	1010 (1311)	202 (312)	41 (73)	0.0399	0.0161	0.0234
3 μm	FF57-60	143 (250)	55 (175)	8 (13)	0.2296	0.0485	0.1889
-	RR17-20	9 (15)	7 (11)	6 (11)	0.7381	0.5855	0.7583

### Comparison of different structure types in SiNW biosensors

As described above, nine biosensor structures applied in this study can divided into three types according to pitch. Notably, having pitch means the structure had a number of parallel nanowires between P and N node, and smaller pitch indicated more nanowires in one structure. Oppositely, no pitch means the structure had only a single nanowire. Results of different pitch were displayed in Table 1 and Table 2.

**Table 2:** Comparison of different pitches in three standard sample concentration (1 pM, 1 fM and 0).

	Let-7b	1 pM	1 fM	0	T Test, p value		
		mean (SD)	mean (SD)	mean (SD)	1 pM vs 1 fM	1 pM vs 0	1 fM vs 0
Pitch	FF47-RR20	179 (136)	113 (87)	67 (54)	0.0098	0.000008	0.0071
1 μm	FF47-50	186 (110)	107 (78)	65 (56)	0.0397	0.0016	0.1269
3 μm	FF57-60	188 (180)	113 (95)	60 (41)	0.1688	0.0169	0.0760
-	RR17-20	161 (112)	119 (92)	80 (66)	0.2837	0.0354	0.2361

In general, structures with pitch (FF47-50 and FF57-60) showed better results than those without pitch (RR17-20). Moreover, structures with 1 μm pitch (FF47, FF49 and FF50) show best detection efficacy in both detection of Let-7b and snRNA U6.

## Discussion

After modification of APTES and glutaraldehyde, the poly-SiNW biosensor device can be used to detect standard sample with detection limit of 1 fM. Additionally, mismatch sequence, even one-base mismatched sequence, can be identified from perfectly matched standard samples. Such sensitivity and specificity were comparable to a previous SiNW study[17]. Notably, PNA probe was utilized to attach the SiNW surface in that study. In general, the PNA-functionalized SiNW device is capable of generating higher change than the DNA-functionalized SiNW device does because DNA is negatively charged, thereby decreasing the signal-to-noise ratio. However, considering the much higher cost of PNA probes, we decided to use DNA probes as others did[19–22]. In general, it is better to use low salt buffer in SiNW because high ion concentration may cause contamination. However, low salt concentration may partly result in the disassociation of the DNA-RNA duplex. Importantly, a recent study developed an assay based on microcantilever arrays which can detect the mutation nanomechanically in total RNA samples isolated from melanoma cells by DNA-total RNA hybridization in 0.01 × SSC, without prior PCR amplification and use of labels[23]. Hence, we also tried to detect miRNA by DNA-miRNA hybridization in 0.01 X SSC. Our device, using ssDNA as probe molecules, reached the detection limit as low as 1fM. The high sensitivity of our DNA-functionalized SiNW device may be caused by the improvement of the fabrication of poly-SiNW biosensor, especially by reducing the pitch, and the modification of data analysis. The poly-SiNW biosensor devices used in the current study were manufactured by standard CMOS procedures and the line width of an individual biosensor could be as small as 25 nm and its pitch as small as 1μm. Although in this study the benefits of smaller line width have not been shown, the detection efficacy was improved by reducing the pitch. Furthermore, we also modified the method of data analysis. Previously, Zhang reported that resistance was measured at 0.1V and mean value was calculated from 25 measurements[17, 24]. However, in standard integrated circus study, electronic examination was usually performed in a range of voltage but not at a fixed voltage[20, 25]. From the initial screening, we found the current was increased with the voltage increased, showing a polynomial curve (S3 File). Such curve indicated the resistance of a SiNW biosensor was not a constant. Therefore, we obtained the current-voltage function (cubic function) according to the raw curve, and then obtain the conductance-voltage function by derivation. Thus AUC in a certain range (0–5 v) represented the conductance of the sample and the ratio of AUC after and before hybridization indicated the resistance change (R/R_0_). This measurement and analysis could obtain more information and better understanding of the resistance change.

Beside standard miRNA samples, this poly-SiNW biosensor device was also used to detect snRNA U6 in total RNA extracted from HepG2 cells. The detection limit reached as low as 0.2 μg/mL and the R/R0 results displayed well linear relationship with concentration. To date, it remains a challenge to detect a target miRNA using SiNW device in total RNA sample. A recent study tried to detect miRNA-122a in the presence of a million-fold excess of total RNA. However, here miRNA-122a was still standard sample and total RNA was extracted from yeast and added to miRNA-122a to make an artificial mixture[25]. For the first time we directly examined snRNA U6 in real biological samples. Finally snRNA U6 in total RNA at a concentration of 0.2 μg/mL could be detected. We compared the results with qPCR, the routine detecting method for miRNAs (S4 File). The results show that qPCR can detect U6 at least from 0.02 μg/mL total RNA. In this aspect, qPCR seems more sensitive than SiNW biosensors currently. However, the process of qPCR contains more than fifteen heat cycles for target amplification and data are obtained through fluorescence and other labels. In addition, there are strict requirements for samples in miRNAs detection with qPCR. That may limits its application in clinic, as clinical samples such as serum and other body fluid always consist of complex ingredients. In contrast, SiNW biosensor could detect miRNA without label and amplification, which may make it promising in future clinical practice.

Among the three types of SiNW, structures with 1 μm pitch (FF47, FF49 and FF50), had the best efficacy, probably due to more nanowires contained in the fix-sized space of a biosensor with 1 μm pitch. Oppositely, structures without pitch (RR17, RR19 and RR20) showed relatively lower efficacy, mostly because of a single silicon nanowire alone existing in each biosensor of this type. Since all the detection data depend on the single silicon nanowire alone, this type of structure had more probability to fail to collect data if the single nanowire cracked. For structures with multiple nanowires, others can still work in case one nanowire cracked. Considering pitch is an important factor influencing the efficacy of SiNW biosensors, future study may seek to improve the SiNW device by reducing the pitch.

Although our poly-SiNW device showed promising results, some shortcomings should be taken into consideration. In this study, each biosensor structure was unique in a SiNW chip and the final detection data were combination of data from different biosensors. That can explain why the standard deviation values were great in detection of standard sample and U6 in total RNA. This study tried to screen out some structures with higher detection efficacy. In later research, devices will be designed to integrate large scale of selected structures. Thus, raw data obtained in one experiment will be multiple and analysis could be improved, providing stronger evidence for the application of the poly-SiNW device.

In general, the poly-SiNW device can detect miRNA directly and quickly with good sensitivity and specificity. Importantly, such poly-SiNW FET device could be developed by standard CMOS technique in nano-scale patterns. This device may have a bright prospect for clinical application.

## Supporting Information

S1 Fig Photo of SiNW through transmission electron microscopy. (TIF)

S1 File Design of poly-SiNW biosensors. A typical 12-inch wafer, which includes 70–80 chips (Figure A). One single chip of wafer, which is comprise of about 400 biosensors (Figure B). A single SiNW biosensor observed under microscopy (Figure C).
(TIF)

S2 File Chemical modification of SiNW. SiNW before chemical modification (Figure A). After modification of 2% APTES, bending vibration of N-H is enhanced in 1650 cm^-1^ (Figure B). After modification of 1.25% glutaraldehyde, stretching vibration of C-O is enhanced in 1730 cm^-1^ (Figure C).
(TIF)

S3 File Linear plot of current and voltage. Well linear relationship between current and voltage (Figure A). Poor linear relationship between current and voltage (Figure B).
(TIF)

S4 File qPCR detection of snRNA U6 from HepG2. Total RNA concentrations are 2, 0.8, 0.2, 0.1, 0.02, 0 μg/mL. qPCR of snRNA U6 (Figure A). qPCR of miR-21 (Figure B).
(TIF)

S1 Table Sequences of standard sample and probe. (DOCX)

S2 Table Comparison of nine SiNW structures. (DOCX)
