# Supplementary figures and images for: Label-Free Direct Detection of miRNAs with Poly-Silicon Nanowire Biosensors
Source: PLoS One. 2015 Dec 28;10(12):e0145160. doi: 10.1371/journal.pone.0145160 (PMC4692481; doi:10.1371/journal.pone.0145160)

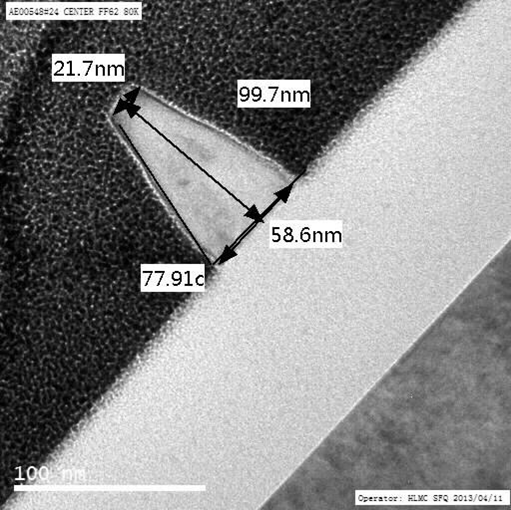

Supplement: S1 Fig — (TIF) [file pone.0145160.s001.tif]

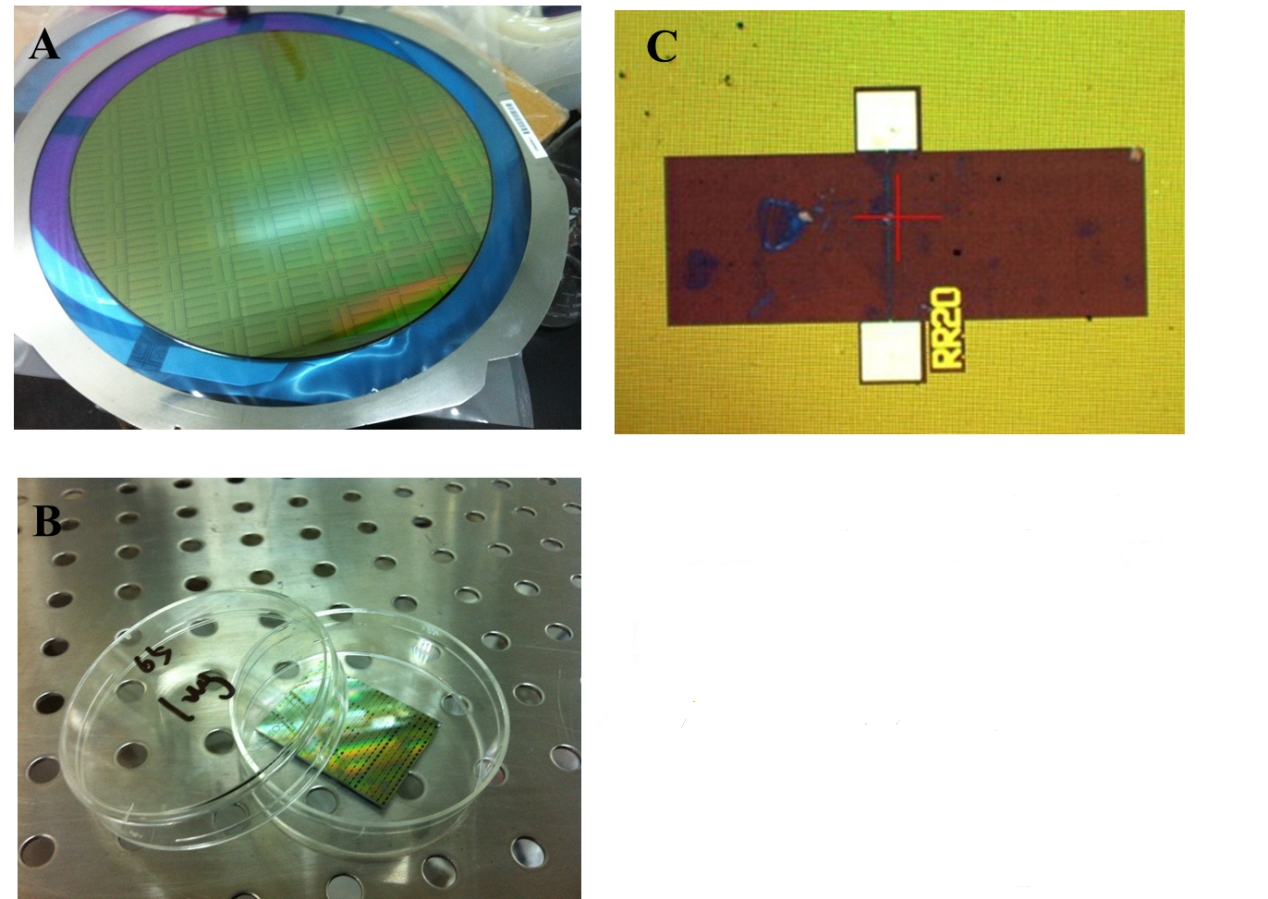

Supplement: S1 File — A typical 12-inch wafer, which includes 70–80 chips (Figure A). One single chip of wafer, which is comprise of about 400 biosensors (Figure B). A single SiNW biosensor observed under microscopy (Figure C). (TIF) [file pone.0145160.s002.tif]

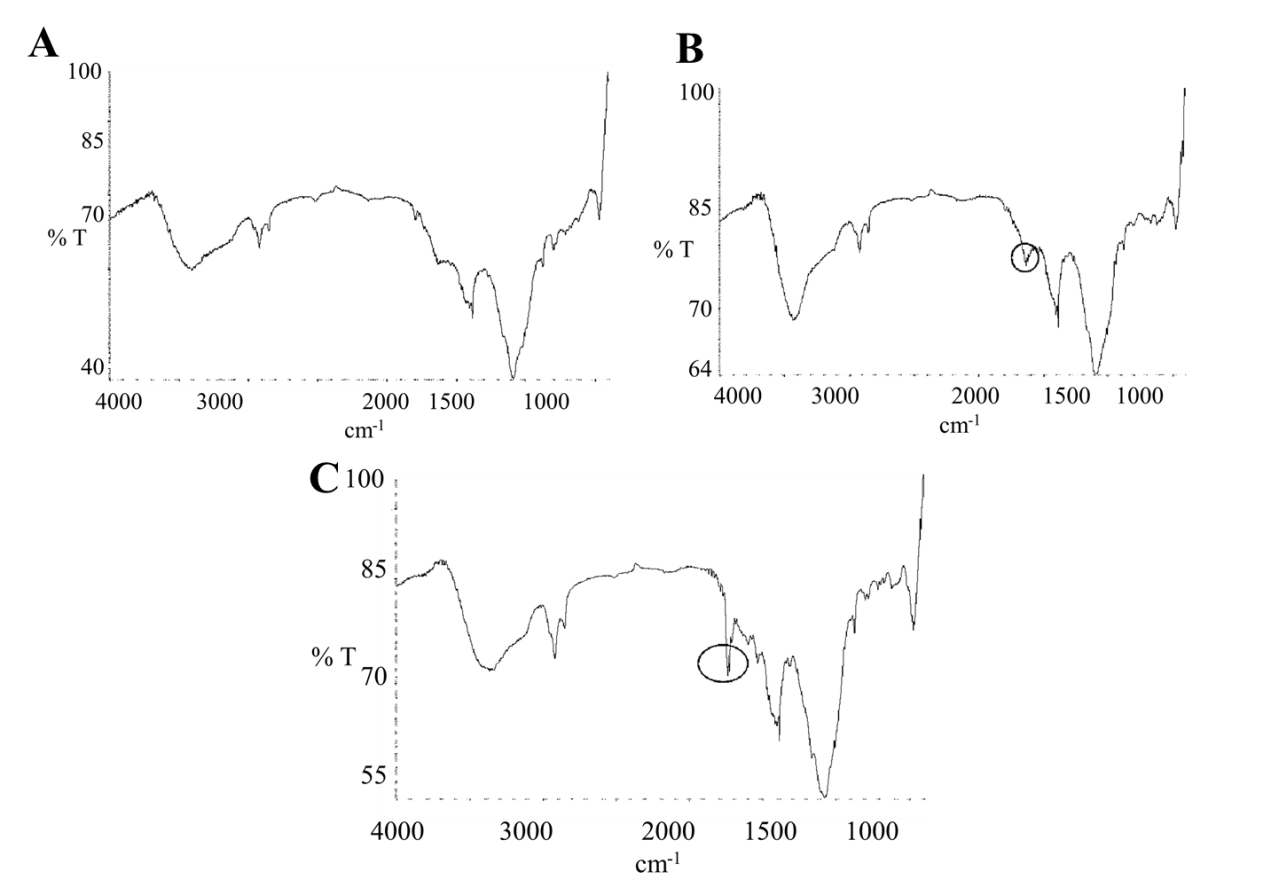

Supplement: S2 File — SiNW before chemical modification (Figure A). After modification of 2% APTES, bending vibration of N-H is enhanced in 1650 cm-1 (Figure B). After modification of 1.25% glutaraldehyde, stretching vibration of C-O is enhanced in 1730 cm-1 (Figure C). (TIF) [file pone.0145160.s003.tif]

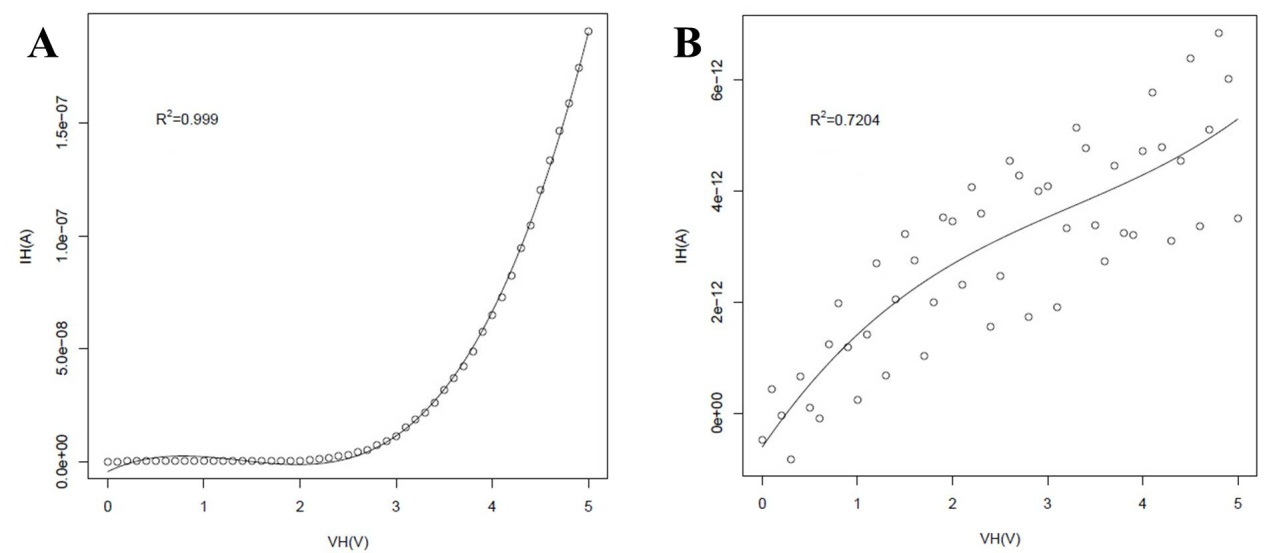

Supplement: S3 File — Well linear relationship between current and voltage (Figure A). Poor linear relationship between current and voltage (Figure B). (TIF) [file pone.0145160.s004.tif]

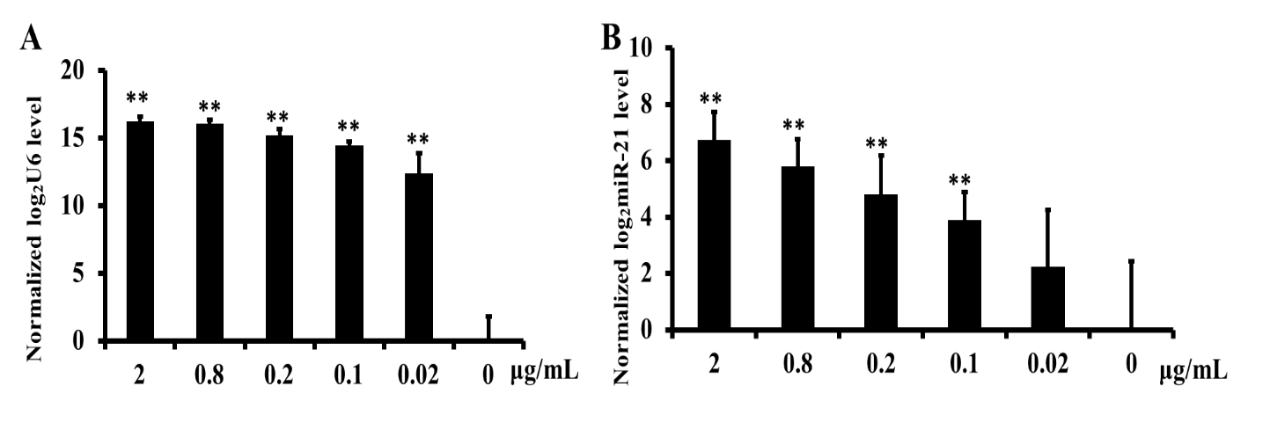

Supplement: S4 File — Total RNA concentrations are 2, 0.8, 0.2, 0.1, 0.02, 0 μg/mL. qPCR of snRNA U6 (Figure A). qPCR of miR-21 (Figure B). (TIF) [file pone.0145160.s005.tif]
