# Supplementary material for: Label-Free Direct Detection of miRNAs with Poly-Silicon Nanowire Biosensors
Source: PLoS One. 2015 Dec 28;10(12):e0145160. doi: 10.1371/journal.pone.0145160 (PMC4692481; doi:10.1371/journal.pone.0145160)
Supplement: S1 Table — (DOCX) [file pone.0145160.s006.docx]

**S1Table.** Sequences of standard sample and probe.

| Standard sample and probe | Sequence |
| --- | --- |
| Let-7b | 5’-UGAGGUAGUAGGUUGUGUGGUU-3’ |
| Let-7c | 5’-UGAGGUAGUAGGUUGUAUGGUU-3’ |
| Mismatch (MM) | 5’-AUGCAUGCAUGCAUGCAUGCAA-3’ |
| Let-7b probe | 5’-AACCACACAACCTACTACCTCA-3’ |
| snRNA U6 probe | 5’-TGCTAATCTTCTCTGT-3’ |
