# Supplementary material for: Label-Free Direct Detection of miRNAs with Poly-Silicon Nanowire Biosensors
Source: PLoS One. 2015 Dec 28;10(12):e0145160. doi: 10.1371/journal.pone.0145160 (PMC4692481; doi:10.1371/journal.pone.0145160)
Supplement: S2 Table — (DOCX) [file pone.0145160.s007.docx]

**S2 Table**. Comparison of nine SiNW structures.

| Structures | Width (nm) | Pitch (μm) |
| --- | --- | --- |
| FF47 | 65 | 1 |
| FF49 | 115 | 1 |
| FF50 | 150 | 1 |
| FF57 | 65 | 3 |
| FF59 | 115 | 3 |
| FF60 | 150 | 3 |
| RR17 | 65 | / |
| RR19 | 115 | / |
| RR20 | 150 | / |

Width, the width of a single silicon nanowires. Pitch, the width between two parallel silicon nanowires. Since RR17, RR19 and RR20 contains only one single nanowire, they do not have pitch.
